# Structural Transformation and Functional Improvement of Potato Protein–Gallic Acid Conjugates: Multispectroscopy and Molecular Dynamics Simulations

**DOI:** 10.3390/foods15030556

**Published:** 2026-02-04

**Authors:** Zhenjing Huang, Jiahao Luo, Xiaoyun Fei, Deming Gong, Xing Hu, Guowen Zhang

**Affiliations:** State Key Laboratory of Food Science and Resources, Nanchang University, Nanchang 330047, China; 7901120097@email.ncu.edu.cn (Z.H.); ncuspyluojiahao@163.com (J.L.); f_xiaoyun0813@163.com (X.F.); dgong01@gmail.com (D.G.); hx0726@ncu.edu.cn (X.H.)

**Keywords:** potato protein, gallic acid, covalent interaction, molecular dynamics simulation, functional characteristics

## Abstract

Covalent modification of polyphenols effectively enhances functional properties of proteins. This study conjugated potato protein isolate (PPI) with gallic acid (GA) via an alkaline method to investigate structural and functional alterations. Successful conjugation was confirmed by a significant decrease in free amino and sulfhydryl groups, coupled with a marked increase in total phenolic content. Multispectroscopic analyses indicated a loosening of the secondary structure and notable changes in the tertiary conformation. Molecular dynamics (MD) simulations corroborated these findings, revealing that GA conjugation induced conformational expansion, improved structural stability, and enhanced surface hydrophilicity. These structural modifications led to substantial functional improvements in the PPI-GA conjugates, including enhanced dispersion stability, improved emulsifying performance, strengthened antioxidant activity, and increased thermal stability. This research may provide effective strategy and technical support for the improvement of functional properties of PPI and expand its application in the food industry.

## 1. Introduction

Plant proteins, characterized by their low cholesterol and saturated fat content, have gained increasing attention as sustainable and health-promoting dietary sources. Among them, potato protein isolate (PPI), a by-product of starch processing, represents an underutilized resource with considerable potential. Compared with some animal protein or other plant protein sources, PPI has relatively low production costs. PPI boasts a high-quality amino acid profile comparable to egg protein, excellent digestibility, and a low risk of triggering allergic reactions, making it superior to proteins from many other crops [1]. Furthermore, PPI exhibits good functional properties, including emulsifying, foaming, and gelling capabilities [2]. However, the numerous challenges faced by PPI, such as insufficient emulsion stability, heat stability and antioxidant capacity, have restricted its application in the complex food systems.

Researchers have tried various modification methods to overcome the functional limitations of native PPI. Both physical and enzymatic methods can effectively improve the functional properties of PPI, such as its emulsion stability [3]. However, physical treatments may induce unpredictable alterations in protein structure, while enzymatic modification, despite its mild conditions, often generates undesirable flavors from the breakdown products. In recent years, covalent conjugation with polyphenols has emerged as a green and effective strategy to enhance the functional properties of proteins. Compared with non-covalent complexes, the formation of covalent conjugates and their influence on protein structure are irreversible, thus helping improve the physicochemical properties of the composite system through stable chemical bonds. For instance, conjugates of whey protein with epigallocatechin gallate (EGCG) showed improved emulsifying and foaming properties [4], while ovalbumin-catechin conjugates enhanced the oxidative stability of fish oil emulsions [5]. Gallic acid (GA) is a natural polyphenol with potent antioxidant and biological activities [6]. Compared with monophenol small molecules, its reactive activity and antioxidant capacity are stronger. Coupled with small molecular weight and low spatial resistance, it has the potential to become an efficient protein graft modifier. Successful conjugation of GA with proteins like moringa protein and whey protein has been reported to significantly improve their solubility, emulsification and thermal stability [7]. These results demonstrate the functional benefits conferred by protein–polyphenol covalent conjugation. However, the underlying molecular mechanisms, such as conformational dynamics and interaction energetics, need to be further explored by advanced research tools capable of probing protein dynamic structures at the atomic level [8].

Molecular dynamics (MD) simulation serves as a powerful computational tool to bridge this knowledge gap, offering atomic-level insights into protein structural dynamics and interaction energetics that are challenging to capture with experimental techniques alone [9]. MD simulation can explain the structural characteristics of protein–polyphenol covalent conjugates at the atomic level, including secondary structure transition [10], stability change, and binding energy [11]. Additionally, MD simulation can form a strong complement to experimental characterization methods. It is worth noting that the application of MD in food protein–polyphenol conjugates is still emerging [12]. Waqar et al. [13] used MD and metadynamics (MTD) simulations for the first time to investigate the structure–function relationship in β-lactoglobulin covalently modified by 4-methylcatechol. On this basis, this study further applied the computational method to the PPI-GA covalently coupled object system, expanding the methodological depth in plant protein research.

Therefore, this work systematically evaluated how covalent conjugation with GA at varying concentrations altered the properties of PPI under alkaline preparation conditions. Structurally, changes in PPI were analyzed by spectroscopic techniques and further elucidated through MD simulations using an innovatively constructed CMpatatin model, which simulated site-specific GA conjugation at 11 lysine residues on patatin, the major protein component of PPI. The functional modifications induced by GA grafting, including surface hydrophobicity, emulsification properties, thermal stability and oxidation resistance, were further explored. This multi-scale research method can not only fill the research gap of PPI-GA conjugates, but also establish a complete cognitive chain from atomic movement to macroscopic function. At the same time, it may provide effective strategies and technical support for enhancing PPI’s performance and expanding its applications in the food industry.

## 2. Materials and Methods

### 2.1. Materials

PPI (purity ≥ 90%) was supplied by Ruimao Biological Co., Ltd. (Xi’an, China). Folin–Ciocalteu reagent and dialysis bag (MWCO: 3500 Da) were purchased from Yuanye Biological Co., Ltd. (Shanghai, China). GA (purity ≥ 99%) was obtained from Merck (Shanghai, China), 5,5-dithiobis-(2-nitrobenzoic acid) (DTNB), o-phthalaldehyde (OPA), L-lysine, 8-Anilino-1-naphthalenesulfonic acid (ANS), DPPH (1,1-diphenyl-2-picrylhydrazyl) and ABTS (2,2′-azino-bis(3-ethylbenzothiazoline-6-sulfonic acid) were obtained from Aladdin Reagent Co., Ltd. (Shanghai, China). Sodium tetraborate, glycine, potassium ferricyanide, and trichloroacetic acid were purchased from Sinopharm Chemical Reagent Co., Ltd. (Shanghai, China). Tris, urea, potassium persulfate, SDS-PAGE kit, prestained low molecular weight protein marker, and loading buffer were supplied by Solarbio Science & Technology Co., Ltd. (Beijing, China). β-Mercaptoethanol was obtained from Shanghai Ruitai Biological Technology Co., Ltd. (Shanghai, China). EDTA was sourced from Xilong Scientific Co., Ltd. (Shantou, China). Ferric chloride (FeCl_3_) was purchased from Shanghai Shiyi Chemical Reagent Co., Ltd. (Shanghai, China). Methanol and glacial acetic acid were also obtained from Xilong Scientific Co., Ltd. (Shantou, China).

### 2.2. Preparation of PPI-GA Covalent Conjugates

PPI was treated following the method of Nimaming et al. [14] with slight modifications. The potato protein isolate powder was dissolved in ultrapure water (1 wt%) and then incubated at 4 °C overnight, after the solution was centrifuged at 7000 rpm for 15 min, the supernatant was collected and freeze-dried for 48 h before collecting the solid powder. Then, the PPI-GA covalent complex was prepared by the alkaline method. Briefly, the freeze-dried PPI was redissolved in ultrapure water to a concentration of 10 mg/mL. This PPI solution was then mixed at a 1:1 (*v*/*v*) ratio with GA solutions of different concentrations (0.4, 0.8, 1.2, 1.6 and 2.0 mg/mL). The pH of the PPI and GA solutions was adjusted to 9.0 using 1 M NaOH, and the mixture pH was monitored at the beginning and end of the reaction. No significant drift was observed. Then, the mixed solution was transferred to a dialysis bag and dialyzed at 4 °C for 48 h. The dialysate was freeze-dried with a vacuum freeze dryer for 48 h to obtain covalent complexes of PPI-GA called PPI-GA0.4, PPI-GA0.8, PPI-GA1.2, PPI-GA1.6 and PPI-GA2.0.

### 2.3. Sodium Dodecyl Sulfate–Polyacrylamide Gel Electrophoresis (SDS-PAGE)

The molecular mass of the covalent conjugates was determined via SDS-PAGE based on Chen et al. [15] with slight modifications. Briefly, electrophoresis was performed using a mini vertical electrophoresis system (Bio-Rad, Shanghai, China) with a 12% separating gel and a 5% stacking gel. Sample preparation involved mixing PPI-GA conjugates (5 mg/mL) with loading buffer at a 4:1 (*v*/*v*) ratio, denatured at 100 °C for 5 min, and then 10 μL of the prepared sample and marker (10–180 kDa) were loaded into each lane. Electrophoresis was conducted at a constant voltage of 180 V for approximately 30 min. After electrophoresis, the gel was stained with Coomassie Brilliant Blue R-250 for 30 min and subsequently destained with a destaining solution (methanol:glacial acetic acid:water = 5:4:1, *v*/*v*/*v*) until the background was clear and protein bands were distinctly visible.

### 2.4. Measurement of Reactive Groups

The free NH2 content was quantified via the OPA method following the procedure of Liu et al. [16] with slight modifications. The reaction was initiated by adding 100 μL of sample (2 mg/mL) to 2 mL of freshly reconstituted OPA reagent [containing 40.0 mg OPA, 1.0 mL absolute methanol, 2.5 mL 20% (*w*/*w*) SDS, 25 mL sodium tetraborate (0.1 mol/L) and 100 μL β-mercaptoethanol]. After incubation at 35 °C for 2 min, the absorbance at 340 nm was measured using a microplate reader (Varioskan LUX^TM^, Waltham, MA, USA). The calibration curves were drawn using different concentrations of lysine solutions (0, 0.2, 0.4, 0.6, 0.8, 1 mg/m L) to determine free amino content. The grafting degree (DG) of the conjugate was obtained with the following formula:

(1) $$\mathrm{DG}\left(\%\right)\;=\;\frac{\left(c_{0}-c_{t}\right)}{c_{0}}\;\times \;100\;$$

In the formula, c_0_ is the content of free amino group of PPI, and c_t_ is the content of free amino group after the reaction of PPI and GA.

The effect of GA covalent binding on free sulfhydryl groups of PPI was evaluated according to Wang et al. [17] with modifications. Briefly, 10 mg of the PPI-GA conjugate was dissolved in 5 mL of Tris-Glycine buffer (0.086 M Tris, 0.09 M glycine, 0.004 M EDTA, 8.0 M urea, 0.5% *w*/*v* SDS, pH 8.0). A 20 µL aliquot of 4.0 mg/mL 5,5′-dithiobis-(2-nitrobenzoic acid) (DTNB) solution was added. The mixture was vortexed and incubated at 25 °C for 30 min in the dark. The absorbances of both the sample blank (identical mixture without DTNB) and the reagent blank (buffer with DTNB but no protein) were subtracted from the sample readings. The absorbance at 412 nm was then measured. The sulfhydryl content of the samples was calculated according to the following formula:

(2) $$\mathrm{Sulfhydryl}\;\mathrm{content}\;\left(\mu \mathrm{mol}/g\right)\;=\;\frac{{(10}^{6}/1.36\;\times {\;10}^{4})\;\times \;A_{412}}{c}$$

where *A*_412_ is the absorbance at 412 nm, 1.36 × 10^4^ is the molar extinction coefficient (L·cm·mol^−1^), and *c* is the mass concentration of protein (mg·mL^−1^).

Changes in GA content after the protein reaction were measured according to the Folin–Ciocalteu method. Briefly, 0.5 mL of PPI-GA conjugate (1 mg/mL) was mixed with 2.5 mL of Folin–Ciocalteu reagent (0.2 M) and allowed to react for 5 min. Then, 2 mL of 7.5% (*w*/*v*) Na_2_CO_3_ was added to the mixture. After vortexing, the reaction proceeded in the dark at room temperature for 2 h, and the absorbance at 760 nm was measured. To account for any potential interference from the protein matrix, a control sample of native PPI (at an equivalent concentration) was processed identically, and its absorbance was subtracted from that of the conjugate samples. A standard curve was prepared using gallic acid solutions at concentrations of 0, 0.01, 0.02, 0.04, 0.08, and 0.1 mg/mL.

### 2.5. Fluorescence Spectra

Fluorescence spectra were recorded using an F-7100 fluorescence spectrometer (Hitachi, Tokyo, Japan). Sample solutions of PPI and PPI-GA conjugates were prepared by dissolving the corresponding powders in ultrapure water to a final concentration of 0.4 mg/mL. All measurements were performed in triplicate. The intrinsic fluorescence spectra were acquired with excitation and emission slit widths set at 2.5 nm. The measurement parameters included an excitation wavelength of 280 nm with emission scanning from 300 to 500 nm. Synchronous fluorescence spectra were acquired at wavelength intervals (Δλ) of 15 and 60 nm, respectively. Three-dimensional fluorescence spectra were acquired under the following conditions: excitation and emission ranges of 200–600 nm with a scan speed of 1200 nm/min [7]. Ultra-pure water without sample was used as a blank control to record the fluorescence intensity change of the sample.

### 2.6. UV Absorption Spectra

The ultraviolet absorption of PPI and PPI-GA conjugates (0.5 mg/mL) was measured between 200 and 400 nm using an ultraviolet spectrophotometer (UV-2600, Shimazu, Kyoto, Japan).

### 2.7. FTIR Spectra

FTIR spectra were acquired using a Nicolet 5700 spectrometer (Thermo Fisher Scientific, Waltham, MA, USA). Pure KBr was used as a blank background, and an appropriate amount of samples was mixed with KBr (1:100), ground into powder and pressed into small pieces. The spectral acquisition covered the 4000–400 cm^−1^ region with the resolution set to 4 cm^−1^. The results were derived using OMNIC 9.7.46 software.

### 2.8. Circular Dichroism (CD) Spectra

Spectrograms of PPI-GA conjugates dissolved in ultrapure water at a concentration of 0.2 mg/mL were recorded using the MOS 450 CD spectrometer (Bio-Logic, Claix, France) in the wavelength range of 190–240 nm at 25 °C. The spectrum of ultrapure water was recorded under identical conditions and subtracted from all sample spectra. DichroWeb (http://dichroweb.cryst.bbk.ac.uk/html/servererror.shtml, last accessed on 25 September 2025) was used to estimate the secondary structure content of PPI-GA conjugates. The analysis employed the SELCON3 algorithm with reference set 4, which was optimized for analysis in the 190–240 nm spectral range.

### 2.9. MD Simulations

MD simulations were conducted referring to the method described by Fei et al. [18], with some modifications. Briefly, the amino acid sequence of patatin protein was retrieved in the NCBI database (PDB number: 4PK9). The tertiary structure of patatin was predicted by the AlphaFolad 3 program, and the protein model was quality-checked by the SAVES v 6.1 program. Molecular dynamics simulation was performed using AMBER 24. The covalent modification of patatin by GA followed the reaction mechanism reported by Fei et al. [7], in which a carbonyl group in the quinone structure formed by GA oxidation dehydrates and condenses with the free amino group on the amino acid. Based on this mechanism, the CMpatatin model was constructed using PyMol 3.1.0. Non-standard residues were treated according to the tutorial on the AMBER website. The model passed the quality assessment on the SAVES v 6.1 server (Figures S1 and S2).

Refer to the tutorial on the AMBER website regarding the treatment of non-standard residues (https://ambermd.org/tutorials/basic/tutorial5/, accessed on 15 September 2025). First, manually construct the modified non-standard residue, named LGA to distinguish it from the standard residue LYS, and save it as a PDB file. Process this PDB file using the antechamber program in AMBER to generate a Prepin file containing the backbone structure and charge distribution of the non-standard residue. Then, manually prepare an .mc file specifying all backbone atoms of LGA, the atoms to be lost when forming peptide bonds with adjacent amino acids, and the atoms that will connect to adjacent amino acids after peptide bond formation. Subsequently, submit both the Prepin file and the .mc file as inputs to the parmchk2 program to generate an frcmod file containing the force field parameters for the non-standard residue. Any missing force field parameters should be manually supplemented. The protonation states of all titratable residues in the patatin structure were assigned using the pdb2pqr tool (available at: https://server.poissonboltzmann.org/pdb2pqr, accessed on 13 September 2025). To match the experimental conditions, the environmental pH was explicitly set at 9.0 during this assignment process for both the unmodified and covalently modified patatin systems.

Load the tLEaP module and load the ff14SB force field and the GAFF force field, where the former is used for standard protein residues and the latter for non-standard residues. Load the structure files along with the Prepin and frcmod files. Solvate the protein in a cubic box with a buffer distance of 10 Å using the TIP3P water model. To neutralize the simulation system, 13 Na^+^ ions are randomly added by replacing the corresponding water molecules. Long-range electrostatic interactions are calculated using the particle mesh Ewald (PME) method. The SHAKE method is applied to constrain bonds involving hydrogen and heavy atoms. Unreasonable spatial interactions were eliminated by energy minimization of the aqueous solvent and protein respectively, then the aqueous solvent and protein was heated from 0 K to 298 K respectively over a 20 ps time span, followed by a constant temperature, constant pressure equalization of 1 ns duration. Temperature and pressure were controlled using a Langevin dynamics thermostat with a collision frequency of 2.0 ps^−1^ and an isotropic position scaling barostat with a relaxation time of 2.0 ps, respectively. Finally, a MD of 100 ns duration on the entire system was performed at 298 K, 1 bar. After the MD simulation was completed, the MD trajectories were analyzed using the CPPTRAJ program. Free energy landscapes were constructed from Gibbs free energy values calculated with GROMACS scripts (g_sham and xpm2txt.py), mapping free energy (*Z*-axis) as a function of RMSD (*X*-axis) and Rg (*Y*-axis). The DSSP program was used to analyze the changes of α-helix and random crimp content in the MD process; the MD simulation results were visualized, rendered and plotted using PyMol 3.1.0.

### 2.10. Particle Size and ζ-Potential

Samples diluted to 2 mg/mL with ultrapure water were analyzed for particle size and ζ-potential at 25 °C using a Zetasizer Nano instrument (Zetasizer Pro, Malvern, Worcestershire, UK), with a refractive index set at 1.45.

### 2.11. Surface Hydrophobicity

ANS: Fluorescence titration with ANS was employed to investigate alterations in the surface hydrophobicity of PPI following covalent modification. Fluorescence measurements were conducted by incubating PPI-GA conjugate solutions (0.05–1 mg/mL, 2 mL) with 40 μL of 8 mM ANS in the dark for 20 min, followed by intensity detection at 380 nm excitation.

Contact angle: Powder samples of PPI and PPI-GA conjugates were compressed into sheets for contact angle measurement. Then, 4 μL water droplets were deposited on the sample surface. The contact angle (θ) was measured using an interfacial rheometer (OCA25, Dataphysics Instruments, Filderstadt, Germany) and calculated based on the droplet’s geometric profile [19].

### 2.12. Emulsification and Emulsion Stability

The emulsifying activity index (EAI) and emulsifying stability index (ESI) of the PPI-GA conjugates were evaluated following the protocol described by Xu et al. [20]. PPI-GA conjugates (2 mg/mL) were homogenized with corn oil (3:1, *v*/*v*) (10,000 rpm) for 3 min. After 30-fold dilution of homogeneous emulsion (100 μL) in 0.1% (*w*/*v*) SDS at 0 and 10 min, absorbance at 500 nm was measured using a microplate reader (Varioskan LUX^TM^, USA). The values of EAI (m^2^/g) and ESI (min) were calculated as follows:

(3) $$\mathrm{EAI}\left(m^{2}/g\right)\;=\;\frac{2\;\times \;2.303\;\times \;A_{0}\;\times \;D}{c\;\times \;\Phi \;\times \;L\;\times \;10^{4}}$$

(4) $$\mathrm{ESI}\left(\min\right)=\frac{A_{0}}{A_{0}-A_{10}}\times 10\;$$

In this expression, D is the dilution factor; c corresponds to the protein concentration (g/mL); Φ denotes the corn oil volume fraction; A_0_ and A_10_ refer to the absorbance at 500 nm immediately after homogenization and 10 min later, respectively.

### 2.13. Antioxidant Activity

DPPH method: A total of 1 mL of the sample (0.5 mg/mL) was mixed with 1 mL of 0.2 mM DPPH ethanolic solution. The reaction mixture was kept in the dark at 25 °C for 1 h, after which the absorbance was recorded at 517 nm.

ABTS method: A 7 mM ABTS aqueous solution was mixed with a 2.45 mM potassium persulfate solution and incubated in the dark for over 12 h. The ABTS working solution was prepared by diluting the stock with ethanol to an absorbance of 0.70 ± 0.02 (734 nm). Subsequently, 1 mL of sample was reacted with 3 mL of this solution in the dark for 1 h, followed by absorbance measurement at 734 nm. The DPPH/ABTS radical scavenging capacity was calculated as follows:

(5) $$A_{d}\left(\%\right)\;=\;\frac{[A_{0}-(A_{t}-A_{s})]}{A_{0}}\times 100\;$$

A_d_ represents the ability of radical scavenging; A_0_, A_t_ and A_s_ represent the absorbance of blank, 1 h after the reaction and control samples, respectively.

FRAP method: A total of 1 mL of the sample solution (0.25 mg/mL, dissolved in 20% *v*/*v* acetic acid solution) was mixed with 1 mL of potassium ferricyanide solution (1%, *w*/*v*). The mixture was vigorously vortexed and incubated at 50 °C in a water bath for 20 min. Then, 1 mL of 10% (*w*/*v*) trichloroacetic acid was added with thorough mixing. From this, a 1 mL aliquot was transferred and mixed with 3 mL deionized water plus 0.4 mL of 0.1% (*w*/*v*) FeCl_3_ solution. After 20 min reaction at room temperature, absorbance was read at 700 nm.

### 2.14. DSC

Thermal stability was characterized using a DSC250 instrument (TA Instruments, New Castle, DE, USA). Briefly, 3 mg of each sample (PPI or PPI–GA conjugates) was evenly placed in an aluminum crucible, which was then hermetically sealed. An empty aluminum pan served as the reference. The thermal scan was performed from 30 to 180 °C at a constant heating rate of 10 °C/min under a nitrogen atmosphere.

### 2.15. Statistical Analysis

All samples were tested three times, and the results were presented as mean ± standard deviations (SD). All the data were analyzed by ANOVA and Duncan’s test to assess differences between groups using IBM SPSS program (IBM Corp., Armonk, NY, USA, version 26.0). *p* < 0.05 was a significant difference.

## 3. Results

### 3.1. SDS-PAGE

**Figure 1:**
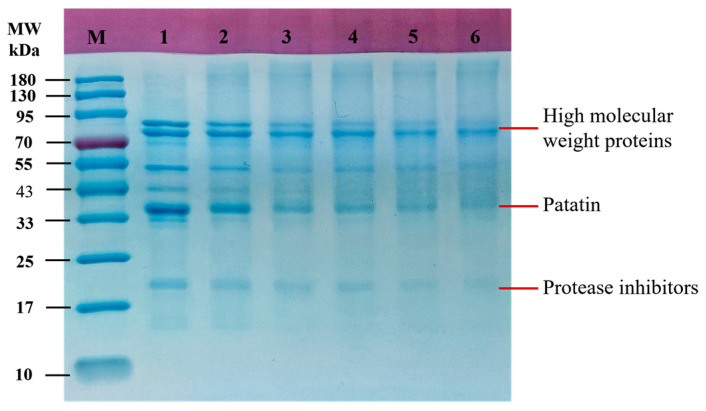
SDS-PAGE profiles of PPI and PPI-GA conjugates at 5 mg/mL. (M: Protein markers; lane 1: PPI; lane 2: PPI-GA0.4; lane 3: PPI-GA0.8; lane 4: PPI-GA1.2; lane 5: PPI-GA1.6; lane 6: PPI-GA2.0).

SDS-PAGE is the most commonly used protein expression analysis technique. Its principle is to separate proteins in electrophoretic gels according to their molecular weight. To verify whether PPI and GA covalently bind successfully, SDS-PAGE analysis was performed first. After covalent treatment, the subunit bands of PPI became shallower (Figure 1). Coomassie Brilliant Blue stains proteins by interacting with basic and aromatic amino acid residues [21]. The attenuated staining suggested that GA modification masked these binding sites, likely because the covalent grafting of GA molecules decreased the accessibility of nucleophilic residues, such as free amino and sulfhydryl groups, to the dye. It could be observed that the conjugate bands showed an upward shift, indicating an increase in the molecular weight of PPI after covalent binding with GA [22]. Considering that SDS and β-mercaptoethanol destroy non-covalent and disulfide bonds in this experiment [23], these results suggested that a covalent conjugate of PPI-GA was successfully formed. Eichhorn et al. [24] found similar results when PPI was covalently bound to pectin.

### 3.2. Measurement of Reactive Groups

**Figure 2:**
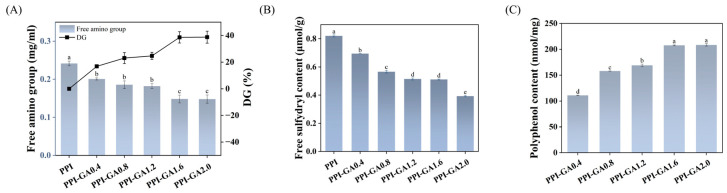
Contents and grafting degree of free amino groups (**A**), free sulfhydryl groups (**B**) and GA content (**C**) in PPI and PPI-GA conjugates. Different letters indicate significant differences in the results (*p* < 0.05).

The free amino group content and the DG serve as reliable indicators for evaluating the extent of covalent conjugation. As the GA concentration increased from 0.4 to 2.0 mg/mL, the free amino group content exhibited a concentration-dependent decrease, falling from 0.24 to 0.15 mg/mL (Figure 2A). This reduction (38.74%) was consistent with the decline in free amino groups observed in peanut protein–gallic acid conjugates under similar alkaline conditions, as reported by Chen et al. [25]. Given that SDS and β-mercaptoethanol used in this experiment disrupted non-covalent interactions and disulfide bonds, the decrease in free amino groups suggested that the ε-amino groups of lysine residues formed covalent bonds with GA [17]. Under alkaline conditions (pH 9.0), polyphenols are oxidized to o-quinones in the presence of reactive oxygen species, while amino groups in proteins become deprotonated, enhancing their reactivity [26]. This promotes a nucleophilic addition reaction, leading to an increased DG and a corresponding reduction in free amino groups [25].

Free sulfhydryl groups in proteins are also highly reactive. As shown in Figure 2B, compared with untreated PPI, the free sulfhydryl content in GA-grafted PPI decreased markedly. At 2.0 mg/mL GA, the free sulfhydryl content dropped notably to 0.73 μmol/g, a trend comparable to that observed in hordein–gallic acid adducts [27]. The presence of urea and SDS in the buffer have eliminated disulfide bond interference and accessibility of all free sulfhydry groups [16]. This decline was attributed to the covalent bonding between sulfhydryl groups and polyphenols during the reaction.

Based on the present results, the reduction in both free amino and free sulfhydryl groups can be ascribed to interactions between reactive polyphenol moieties and protein nucleophiles [16]. The hydroxyl groups of polyphenols are their primary reactive sites and play a key role in protein–polyphenol interactions: on one hand, they can engage in weak interactions such as hydrogen bonding with proteins; on the other hand, under alkaline oxidative conditions, these hydroxyls are converted into quinones, which act as cross-linkers and undergo covalent coupling with nucleophilic groups (e.g., amino and sulfhydryl residues) via Schiff-base reactions and Michael addition [28]. Therefore, the pronounced decline in free amino and thiol groups further supports the formation of covalent C–N and C–S bonds in the PPI–GA conjugates.

The total phenol content in the covalent conjugate of PPI-GA was determined by the Folin–Ciocalteu method, which relies on the reducing capacity of polyphenols. As shown in Figure 2C, when the GA concentration was increased to 1.6 mg/mL, the grafted GA content reached a maximum of 207.81 nmol/mg. Because unreacted GA was removed by dialysis, higher GA content reflected a greater degree of conjugation. Further increases in GA concentration did not lead to a significant rise in grafting, suggesting that the GA binding sites on PPI may have become saturated.

### 3.3. UV Absorption Spectra

**Figure 3:**
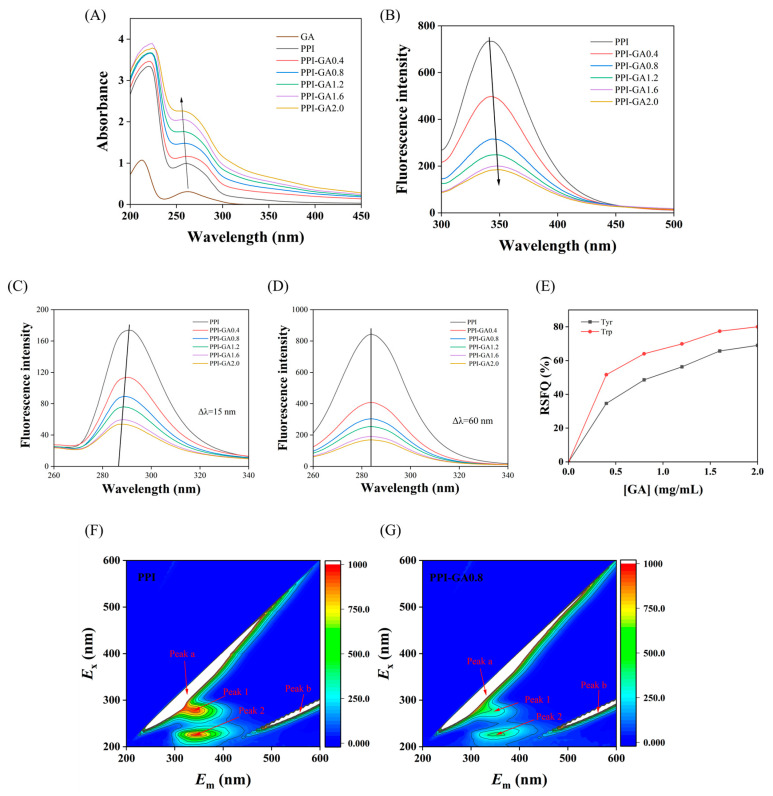
UV absorption spectra (**A**), intrinsic fluorescence spectra (**B**), synchronous fluorescence spectra at Δλ = 15 nm (**C**) and Δλ = 60 nm (**D**), synchronous fluorescence quenching ratio (RSFQ) plots (**E**) and 3D fluorescence spectra (**F**,**G**) of PPI and PPI-GA conjugates.

In the 260–300 nm range, PPI UV absorption is dominated by aromatic residues (Trp, Tyr) via π–π* transitions [29]. All measurements had excellent reproducibility, and the spectra of ultrapure water (used as a solvent) were subtracted to subtract the background. Following covalent GA modification, the conjugates exhibited elevated absorption intensity and a blue shift by 2–6 nm in the maximum absorption wavelength (Figure 3A). This was due to the changes of conformation in the protein and microenvironment around Trp and Tyr residues due to the interaction with GA [30]. Since unreacted GA was removed by dialysis, the observed increase in UV absorption intensity can be attributed to the incorporation of phenolic groups onto the protein [31]. Furthermore, all covalent conjugates displayed a moderate shoulder in the 300–350 nm region, along with a weak, broad absorption tail beyond 350 nm, which was characteristic of covalent bonding between proteins and polyphenol moieties [32].

### 3.4. Fluorescence Spectra

Covalent conjugation between proteins and polyphenols can significantly change fluorescence properties by altering the chromophore microenvironment. Trp, Tyr and phenylalanine serve as the primary fluorophores in proteins, and changes in their chemical surroundings directly affect fluorescence intensity [33]. It was observed that fluorescence intensity decreased and the maximum emission wavelength red-shifted (from 340 nm to 349 nm) after covalent binding of PPI with GA (Figure 3B). This was attributed to the interaction of the oxidized o-quinone structure with the exposed aromatic amino acids in PPI, quenching the excited state energy. At the same time, the interaction between the conjugated structure of GA and the protein increased the degree of conjugation of the chromophore, resulting in a change in the electron cloud density around the fluorophores of PPI. These results are consistent with structural changes in walnut protein–polyphenol covalent complexes [34].

Synchronous fluorescence spectroscopy is more sensitive to environmental changes and can provide detailed information about the microenvironment around the fluorophore, further analyzing the local microenvironment changes. Spectra recorded at Δλ = 15 nm and Δλ = 60 nm reflect microenvironment variations around Tyr and Trp residues, respectively. Fluorescence spectra showed Tyr quenching and a blue-shifted emission maximum upon GA binding (Figure 3C), indicating that GA conjugation altered PPI tertiary structure and transferred Tyr residues to a more hydrophobic environment [35]. Meanwhile, fluorescence intensity of Trp also decreased after covalent binding, though its emission maximum remained unchanged. The synchronous fluorescence quenching ratio (RSFQ = 1 − F/F_0_) of Trp was significantly higher than that of Tyr (Figure 3D,E), indicating that Trp residues experienced more pronounced environmental perturbation. These results supported the conclusion that Trp residues were located closer to the GA modification sites and played a dominant role in PPI fluorescence quenching [36].

To further elucidate the PPI–GA binding mechanism, 3D fluorescence spectroscopy, which provides comprehensive spectral information across all excitation and emission wavelengths, was employed in the study. Two characteristic peaks were observed (Figure 3F,G): Peak a (rayleigh scattering) and Peak b (second-order scattering). Peak 1 and Peak 2 were assigned to fluorescence emissions from Trp/Tyr residues and the polypeptide backbone, respectively [37]. Compared with the control (Figure 3F), the conjugate (Figure 3G) exhibited marked reductions in the intensities of both Peak 1 and Peak 2, confirming that GA induced tertiary structural changes in PPI [38].

### 3.5. FT-IR Spectra

**Figure 4:**
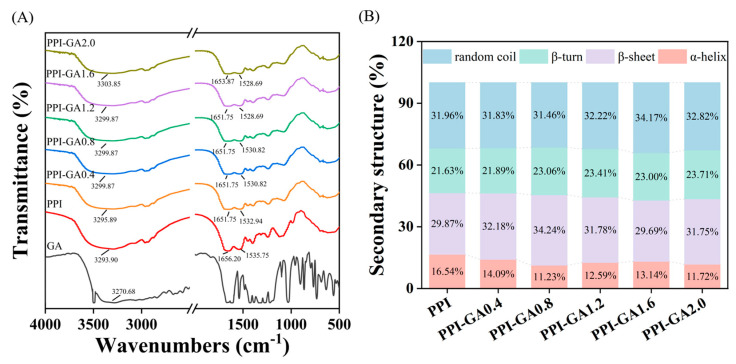
FT-IR spectra (**A**) and secondary structure content (**B**) of PPI and PPI-GA conjugates.

FTIR spectroscopy provides structural information of proteins by detecting the vibrational absorption of chemical bonds, where distinct bonds and functional groups exhibit characteristic peaks in specific wavenumber regions. In PPI, characteristic absorption bands were observed at 1656.20 cm^−1^ for amide I (C=O stretching), 1535.75 cm^−1^ for amide II (N–H bending and C–N stretching), and 3293.90 cm^−1^ for amide A (N–H stretching) [39]. As presented in Figure 4A, covalent binding with GA resulted in shifts in the amide I and amide II peak positions, indicating alterations in the chemical environment of the amide bonds. The spectral data revealed an attenuation in peak intensity, confirming covalent interaction between PPI and GA [40]. Additionally, the amide A band appeared distinctly broadened with variable intensification, implying reinforcement of the hydrogen-bonding network attributable to –OH and –NH stretching vibrations. In phenolic compounds, the O–H stretching vibration typically appears between 3200 and 3600 cm^−1^ [41]. Upon covalent binding, GA’s phenolic hydroxyl groups were oxidized to quinones and reacted with protein functional groups, leading to a decrease in the intensity of the corresponding IR absorption. This reduction reflected both the consumption of phenolic hydroxyls and the altered chemical environment dampening their vibrational modes [42].

### 3.6. CD Analysis

The secondary structure of PPI following covalent modification with GA was analyzed by CD spectroscopy. As shown in Figure 4B, covalent binding with GA resulted in a maximum reduction of 5.31% in the α-helix content. This structural change likely resulted from the disruption of hydrogen-bonding networks in α-helices via polyphenol-amide interactions, combined with covalent bonding to side-chain residues. Such interactions disrupted the original hydrogen-bonding network, leading to a structural transition from α-helix to random coil and β-sheet conformations [43]. Consistent with this mechanism, our results showed a corresponding increase in random coil and β-sheet content. These structural changes suggested a loosening of PPI conformation, characterized by a shift from ordered to more disordered secondary structures. A similar finding was reported in the β-lactoglobulin–EGCG/CA conjugates [44].

### 3.7. MD Simulations

**Figure 5:**
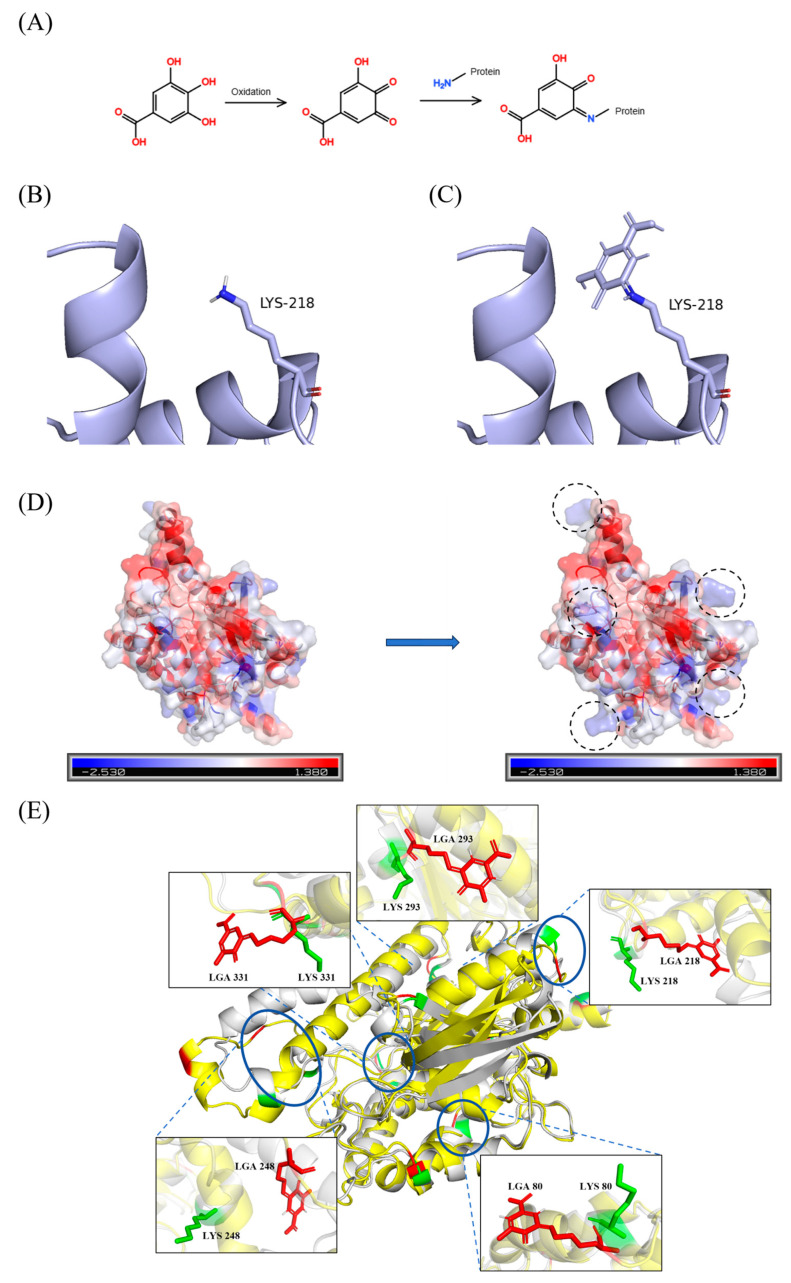
(**A**) A diagram showing the mechanism of covalent interaction between GA and PPI; Lys structure before (**B**) and after (**C**) covalent modification; (**D**) the distribution of surface hydrophobicity before and after GA covalent modification, in which the color represents the relative hydrophobicity of each point on the surface: red represents the hydrophobic region, blue represents the hydrophilic region; (**E**) the contrast between the lowest energy conformation of unmodified patatin and GA covalently modified patatin: gray represents unmodified patatin, yellow represents GA-modified patatin, green represents unmodified amino acid residues, and red highlights covalently modified lysine residues.

**Figure 6:**
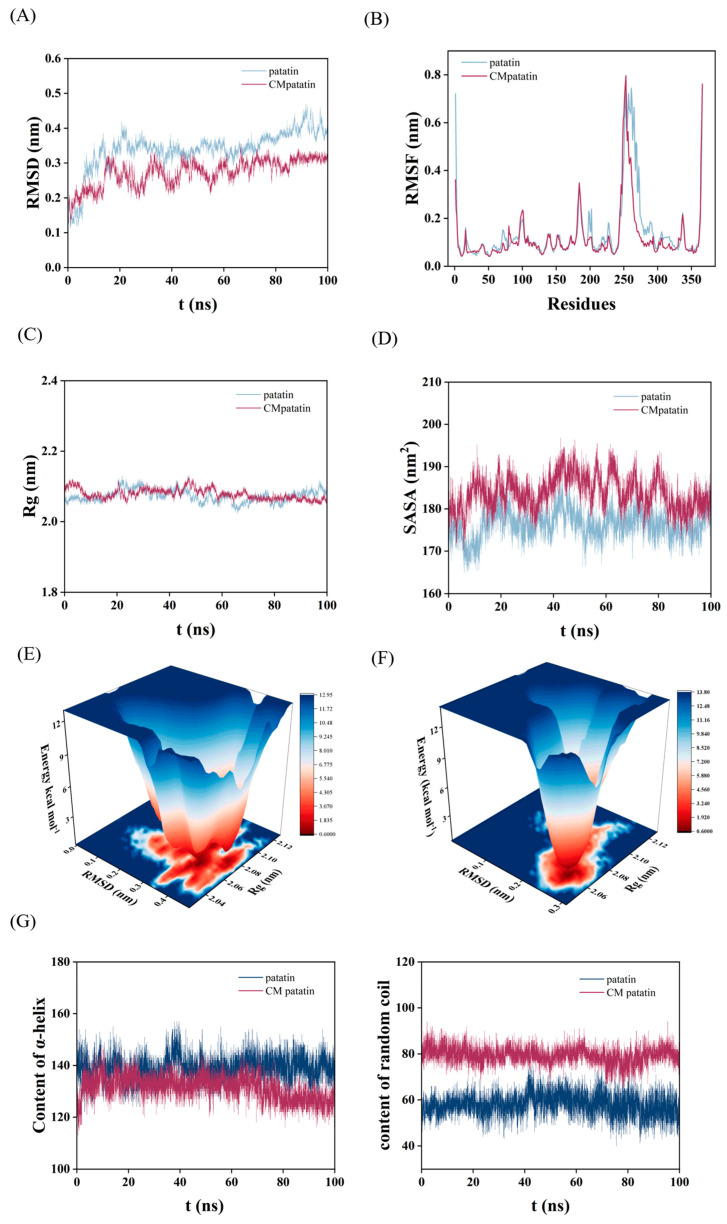
Root mean square deviation (RMSD) throughout the 100 ns MD simulation (**A**), root mean square fluctuation (RMSF) (**B**), time evolution of the radius of gyration (Rg) (**C**) and solvent accessible surface area (SASA) (**D**) for unmodified patatin and GA covalently modified patatin; Gibbs free energy landscape of unmodified patatin (**E**) and GA covalently modified patatin (**F**); secondary structure composition analysis throughout the MD simulation trajectory for unmodified patatin and GA covalently modified patatin as calculated by DSSP (**G**).

Patatin protein is the main component of PPI, because it not only accounts for approximately 40% of the total PPI, but also has good emulsification and interface properties. Since patatin is the primary constituent of PPI and its structural information is complete, the selection of patatin is helpful to the study of its covalent interaction with GA at the atomic level. MD simulation results revealed the regulatory mechanism of GA covalent modification on the conformation of patatin. According to the grafting degree measurements, each patatin molecule is estimated to conjugate with 10 to 15 GA molecules covalently. Based on previous experimental evidence [16], it could be assumed that the protein bound to GA covalently: in the presence of oxygen, the phthaloglucinol structure of GA is oxidized to a highly reactive o-quinone structure. The newly formed o-quinone structure acts as an electrophilic center and is attacked by nucleophilic groups (such as Lys) on the protein to form stable covalent bonds (such as C-N or C-S bonds) (Figure 5A). Analysis of the patatin surface revealed that lysine residues account for the majority of accessible free amino groups. Accordingly, 11 surface-exposed lysine residues (Lys80, Lys108, Lys141, Lys218, Lys248, Lys253, Lys293, Lys331, Lys332, Lys351 and Lys363) were selected as modification sites. Structural comparisons before and after modification (Figure 5B,C) illustrated the local conformational rearrangement upon GA conjugation. The RMSD of the GA-modified patatin decreased significantly relative to the native protein and stabilized after 60 ns with reduced fluctuations (Figure 6A), indicating enhanced structural stability [45]. This stabilization was further reflected in the free energy landscape, where the modified protein transitioned from multiple shallow energy basins to a single deep and smooth energy well (Figure 6E,F). The darkest blue region represented the most thermodynamically stable state, confirming that covalent modification induced a well-defined, stabilized conformation [46].

To characterize structural changes in the lowest free-energy state, we compared the minimum-energy conformations before and after modification (Figure 5E). A marked unfolding of originally ordered α-helical segments was observed around GA modification sites, with these regions adopting more extended and dynamic random coil conformations. DSSP-based secondary structure analysis confirmed a decrease in α-helix content and a concomitant increase in random coil throughout the simulation (Figure 6G), consistent with our CD results. This order-to-disorder transition suggested disruption of the native hydrogen-bonding network and increased structural flexibility, which may facilitate the exposure of functional sites and contribute to functional enhancement.

Despite the overall stabilization, the RMSF of the modified protein decreased only slightly (Figure 6B), indicating that GA conjugation did not strongly restrict side-chain mobility at most residue positions [47]. This finding aligned with the minor changes in the Trp microenvironment observed by synchronous fluorescence analysis. Concurrently, the increases in the Rg and SASA (Figure 6C,D) revealed the conformational expansion of the protein upon modification [48]. This structural shift was also evident from the emergence of blue hydrophilic patches on the protein surface after modification (Figure 5D), suggesting a rise in hydrophilicity resulting from the hydrophilic moieties introduced by GA.

### 3.8. Particle Size and ζ-Potential

**Figure 7:**
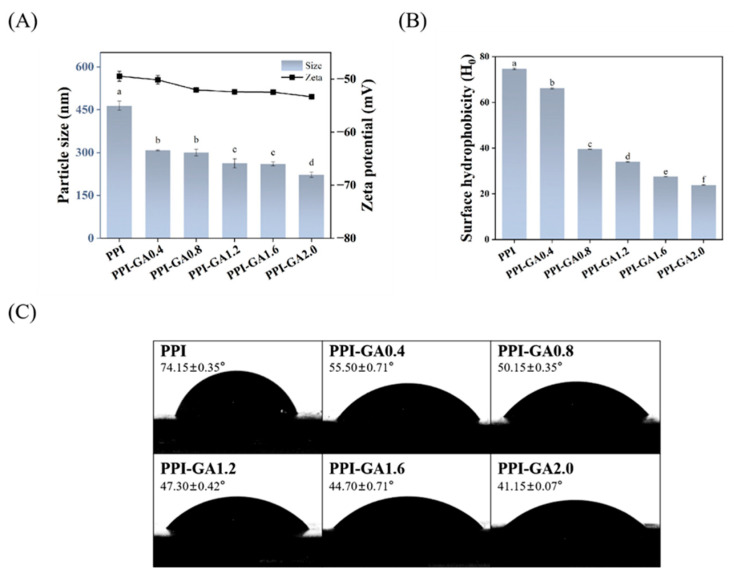
Average particle size and zeta potential (**A**), surface hydrophobicity (**B**) and contact angle (**C**) of PPI and PPI-GA conjugates. Different letters indicate significant differences in the results (*p* < 0.05).

As shown in Figure 7A, the particle size of PPI decreased from 463.85 nm (PPI) to 222.90 nm (PPI-GA2.0) with increasing GA concentration. This reduction may be attributed to the covalent modification of free amino and thiol groups, which limits intermolecular cross-linking and suppresses the formation of large aggregates, consistent with the findings of Yi et al. [49]. In parallel, the absolute ζ-potential value increased with GA concentration. This change resulted from the introduction of GA molecules. At the same time, the covalent binding of GA to PPI induced secondary structural rearrangement, which led to the masking of positively charged groups. This effect, combined with the introduction of anionic galloyl moieties from GA, contributed to the increased absolute ζ-potential value [50]. However, the change of particle size and ζ-potential did not show a strict linear concentration trend, which may be due to the overlap of some numerical differences between samples of different concentrations within the error range.

### 3.9. Surface Hydrophobicity

The surface hydrophobicity of PPI was evaluated using ANS, a hydrophobic fluorescent probe whose fluorescence intensity increases upon binding to hydrophobic regions on the protein surface. A marked decrease in surface hydrophobicity (68.02%) was observed for PPI–GA 2.0 following covalent conjugation with GA (Figure 7B). In addition, the introduction of hydrophilic groups from GA during covalent interaction further enhanced surface hydrophilicity, thereby reducing overall hydrophobicity [51]. This observation was consistent with findings from Wang et al. [52], but contrasted with those of Pi et al. [53]. GA is a small molecule with concentrated hydrophilic groups and low steric hindrance, potentially allowing it to efficiently modify and shield hydrophobic regions on the protein surface. In contrast, larger polyphenols with greater steric bulk may modify the protein surface differently, potentially leading to alternative structural rearrangements that expose hydrophobic domains [54].

To further validate these surface property changes, contact angle measurements were performed. A smaller contact angle indicates stronger surface hydrophilicity and better liquid spreading ability. As depicted in Figure 7C, the contact angle of the PPI–GA conjugates were markedly smaller than that of native PPI (74.15°). At a GA concentration of 2.0 mg/mL, the contact angle attained its lowest value of 41.15°. This reduction was associated with the incorporation of polar groups from GA, which increased the number of hydrophilic moieties on the protein surface [55]. These results were also consistent with those presented in Section 3.2, Measurement of Reactive Groups. Interestingly, a similar trend was also observed in the EWP-TA/GA conjugates [19], further supporting the conclusions of this study. The indirect measurement of the ANS fluorescence probe and the direct characterization of the contact angle confirmed each other, and together showed that the surface properties of PPI and GA covalently bound changed significantly, and the overall hydrophobicity decreased.

### 3.10. Emulsifying Properties

**Figure 8:**
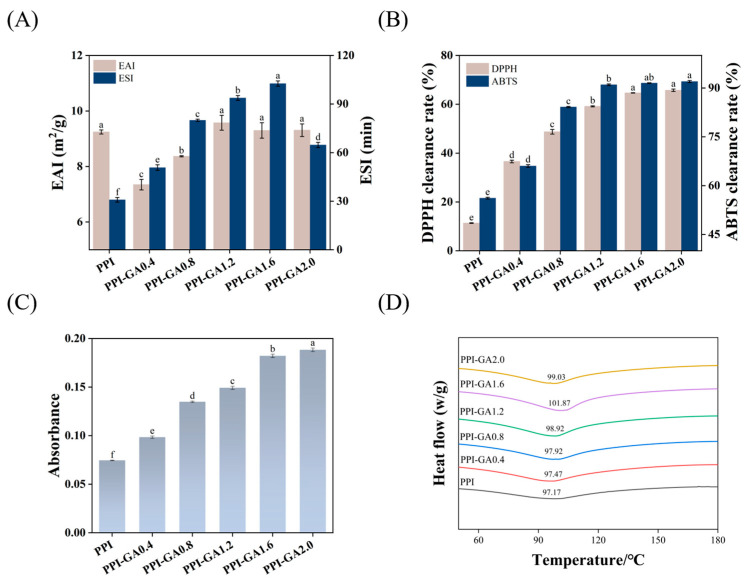
Emulsification and emulsifying stability (**A**), DPPH and ABTS radical scavenging (**B**) and FRAP (**C**) of PPI and PPI-GA conjugates; (**D**) DSC curves of PPI and PPI-GA conjugates. Different letters indicate significant differences in the results (*p* < 0.05).

As primary indicators of emulsification performance, EAI and ESI denote the initial emulsification capacity and long-term stability, respectively. As the GA concentration increased, the EAI of PPI–GA conjugates exhibited a trend of initial decrease followed by recovery (Figure 8A). At low GA concentration, the EAI decreased from 9.24 m^2^/g (PPI) to 7.35 m^2^/g (PPI-GA 0.4). This reduction can be explained by GA molecules occupying key hydrophobic regions on PPI, thereby impairing its interaction with the oil phase, which was supported by the observed decrease in surface hydrophobicity. As the GA concentration further increased, the EAI rebounded. This recovery was attributed to excessive GA promoting intermolecular cross-linking, leading to the formation of a flexible and dispersed network structure, as indicated by the reduction in particle size. Such structural changes facilitate the expansion and rearrangement of PPI at the oil–water interface, thereby partially restoring emulsifying activity [56]. A similar pattern has been reported by Hu et al. [57].

Regarding emulsifying stability, low polyphenol concentrations significantly improved the ESI, which increased from 30.84 min (PPI) to 102.66 min (PPI-GA1.6). This enhancement was due to the stable interface film formed by the enhanced intermolecular forces of proteins [58]. However, when the GA concentration reached 2.0 mg/mL, excessive cross-linking likely produced an overly dense or non-uniform interfacial coating, which caused a decline in the stability [59].

### 3.11. Antioxidant Activity

DPPH and ABTS radical scavenging assays, together with the FRAP method, were utilized to determine the antioxidant capacity of the PPI–GA conjugates. A significant enhancement in radical scavenging capacity was observed for the conjugates relative to native PPI (Figure 8B). When the GA concentration increased to 2.0 mg/mL, the DPPH radical scavenging rate rose to 65.77%, compared to only 11.39% for PPI. This is because GA itself has a phenolic hydroxyl group, which can provide hydrogen atoms for scavenging free radicals and has a high antioxidant activity [60]. More importantly, the exposure of reducing groups within PPI was induced by covalent binding [61]. A similar improving trend was observed in FRAP assay (Figure 8C). At a GA concentration of 2.0 mg/mL, the iron-reducing ability reached its maximum. This concentration-dependent improvement aligns with earlier findings on SPI-TA conjugates [62]. In summary, covalent interaction with GA significantly improved the antioxidant performance of PPI.

### 3.12. Thermal Stability

The thermal stability of a protein refers to its capacity to resist structural changes induced by heating within a given temperature range. DSC can be used to assess this property by measuring the protein’s denaturation temperature [63]. As shown in Figure 8D, the denaturation temperature of covalently modified PPI increased to 101.87 °C, compared to 97.17 °C for the native PPI control. This improvement can be explained by the formation of hydrogen bonds between GA and PPI after covalent binding, which helped stabilize the protein structure. Moreover, at appropriate polyphenol concentrations, covalent modification appeared to shield hydrophobic regions of the protein from water, thereby enhancing thermal stability. A similar enhancement in thermal stability following polyphenol binding has been reported by Liu et al. [64]. In the present study, however, the increase in denaturation temperature was relatively moderate, which may be related to the low molecular weight of GA. By contrast, larger polyphenols with multi-hydroxyl structures often form extensive hydrogen-bonding networks with polypeptide chains and engage in hydrophobic interactions with amino acid residues, leading to more pronounced thermal stabilization [65]. However, further increases in GA concentration did not yield a corresponding improvement in thermal stability. When the GA concentration reached 2.0 mg/mL, the denaturation temperature decreased slightly. This decline may be attributed to the introduction of excessive hydrophilic GA molecules, which altered the hydration layer around the protein and potentially disrupted its structural integrity. In addition, as shown by the CD results, covalent conjugation with polyphenols can reduce the α-helix content of the protein. Such a loss of ordered secondary structure may also contribute to lower thermal stability [66]. Therefore, at higher GA concentrations, over-modification may reduce the thermal stability of the conjugate.

## 4. Conclusions

The structural and functional consequences of covalently modifying PPI with GA were explored using the conjugates synthesized at varying GA concentrations under alkaline conditions. A combination of multi-scale characterization with MD simulations was used to elucidate the structural evolution and functional enhancement of PPI after modification from the atomic to the macroscopic level. The results demonstrated that covalent interaction with GA induced significant structural changes in PPI, including a 5.31% reduction in α-helix content and a transition toward random coil and β-sheet structures. MD simulations provided atomic-level insights, showing decreased RMSD with faster convergence, along with increased Rg and SASA values, indicating the conformational expansion and enhanced flexibility. The covalent modification led to the alteration in the surface properties of PPI with surface hydrophobicity decreasing and the water contact angle declining from 74.15° to 41.20°. Moreover, as GA concentration increased, the particle size of the conjugates decreased from 463.85 nm to 222.90 nm, reflecting improved dispersion stability. These structural modifications collectively contributed to the enhancement of functional properties. Compared with unmodified PPI, the GA conjugates exhibited markedly improved emulsifying stability, antioxidant activity, and thermal stability, demonstrating strong potential for food industry applications. Future research should focus on evaluating the performance of PPI–GA conjugates in real food matrices and exploring the structure–activity relationships between different polyphenol types and protein modification efficacy, thereby facilitating the high-value utilization of PPI.

## Data Availability

The original contributions presented in the study are included in the article, further inquiries can be directed to the corresponding author.

## Supplementary Materials

The following supporting information can be downloaded at: https://www.mdpi.com/article/10.3390/foods15030556/s1. Figure S1 3D-1D Score Map of CM Patatin Model; Figure S2 Ramachandran diagram of CM-patatin model.

## Abbreviations

The following abbreviations are used in this manuscript:

PPI	Potato protein isolate
GA	Gallic acid
CMpatatin	Gallic acid-modified patatin protein
ANS	8-Anilino-1-naphthalenesulfonic acid
SDS	Sodium dodecyl sulfate
DPPH	1,1-diphenyl-2-picrylhydrazyl
ABTS	2,2′-azino-bis (3-ethylbenzothiazoline-6-sulfonic acid)
FRAP	Ferric reducing antioxidant power
DG	Degree of graft
FT-IR	Fourier Transform Infrared
PDI	Polydispersity Index
H_0_	Surface Hydrophobicity
EAI	Emulsifying Activity Index
ESI	Emulsifying Stability Index
DSC	Differential scanning calorimetry
