# Supplementary figures and images for: Structural Transformation and Functional Improvement of Potato Protein–Gallic Acid Conjugates: Multispectroscopy and Molecular Dynamics Simulations
Source: Foods. 2026 Feb 4;15(3):556. doi: 10.3390/foods15030556 (PMC12896807; doi:10.3390/foods15030556)

**Figure S1.** 3D-1D Score Map of CM Patatin Model

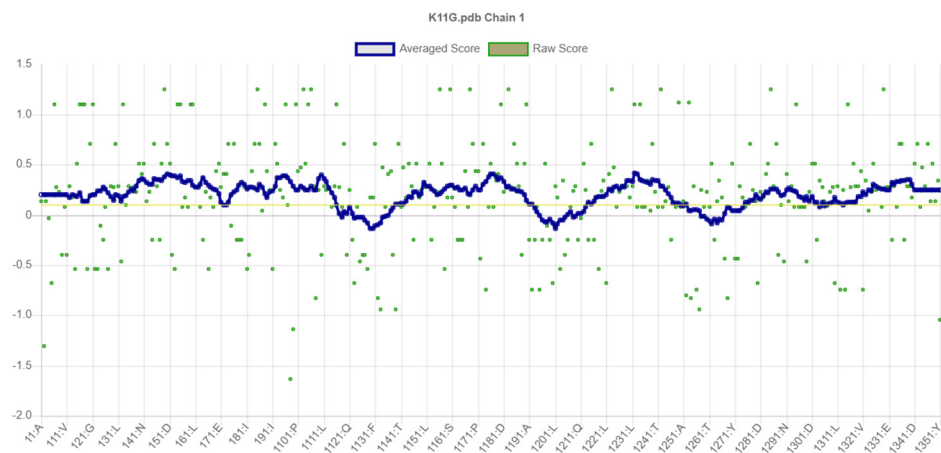

**Figure S2.** Ramachandran diagram of CM-patatin model

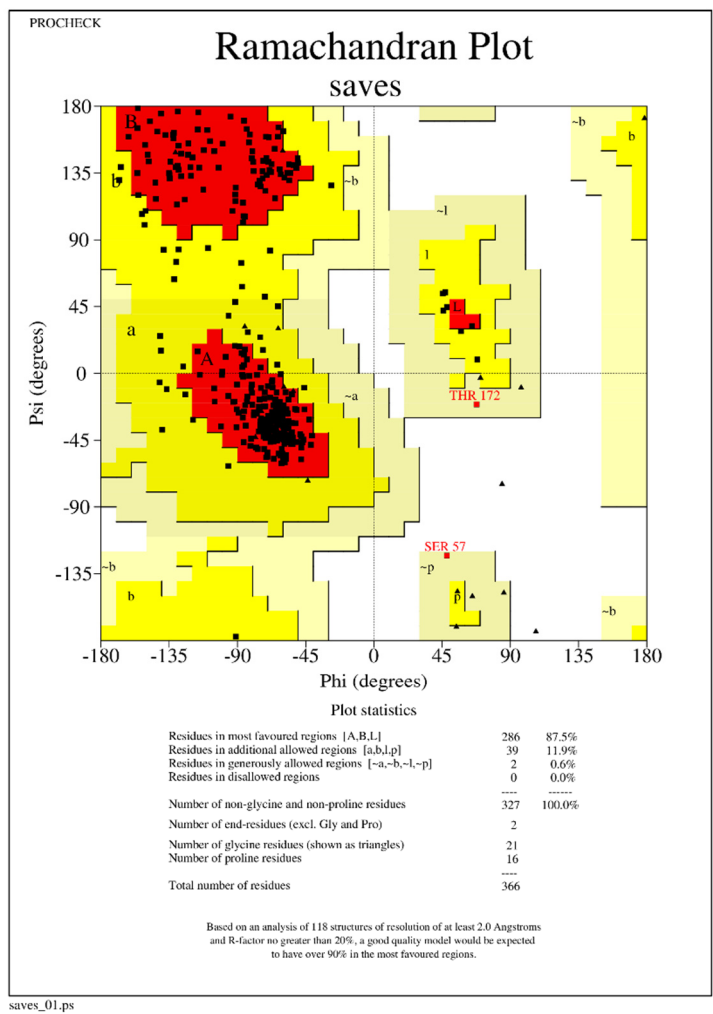

Supplement: Supplementary file 1 [file foods-15-00556-s001.zip › foods-4099400-supplementary.pdf]
